# Breakthroughs in choroid plexus and CSF biology from the first European Choroid plexus Scientific Forum (ECSF)

**DOI:** 10.1186/s12987-024-00546-4

**Published:** 2024-05-21

**Authors:** Laura Pellegrini, Violeta Silva-Vargas, Annarita Patrizi

**Affiliations:** 1 Centre for Developmental Neurobiology, Guys Campus, King’s College London, New Hunt’s House, London, UK; 2 Biozentrum, University of Basel, Basel, Switzerland; 3 Schaller Research Group, German Cancer Research Center (DKFZ), Heidelberg, Germany

**Keywords:** Neurodevelopment, Aging, Organoids, Choroid plexus tumors, Cerebrospinal fluid, Fibroblasts, Neuroepithelium, Cilia, Ventricular system, Epithelial cells

## Abstract

The European Choroid plexus Scientific Forum (ECSF), held in Heidelberg, Germany between the 7th and 9th of November 2023, involved 21 speakers from eight countries. ECSF focused on discussing cutting-edge fundamental and medical research related to the development and functions of the choroid plexus and its implications for health, aging, and disease, including choroid plexus tumors. In addition to new findings in this expanding field, innovative approaches, animal models and 3D in vitro models were showcased to encourage further investigation into choroid plexus and cerebrospinal fluid roles.

## Background: The European Choroid plexus Scientific Forum (ECSF)

In the evolving field of neuroscience, the choroid plexus (ChP) and cerebrospinal fluid (CSF) have transitioned from peripheral elements to key players in our understanding of brain health and disease. The European Choroid plexus Scientific Forum (ECSF) meeting in Heidelberg marked a significant milestone, uniting a group of researchers from across Europe with a shared focus on the ChP and CSF. ECSF stood out as a unique opportunity to bring together a highly diverse and enthusiastic group of researchers, who shared the latest insights on the ChP-CSF system from a broad range of backgrounds ranging from ChP biology and physiology, CSF production and regulation, neurovascular and stromal interactions, neuroimmune communications, ChP diseases, with a major focus on local tumors and state-of-the-art technologies. Emphasising collaboration and exchange of new ideas, the ECSF meeting aimed to create a European network of experts, promoting an environment where innovative thinking and a collaborative spirit could thrive, to ultimately advance the field of ChP and CSF research.

Building on the momentum of the ECSF, there is a strong desire to further develop this initiative into an open and larger, inclusive community. Key objectives include securing funding to support ongoing and future research endeavors, organising regular meetings to maintain the flow of information and ideas, and strengthening scientific communication and links among researchers. This initiative will ensure a sustained and productive collaboration, enabling the community to delve deeper into the biology of the ChP and CSF and its impact on brain health and disease.

**Fig. 1:**
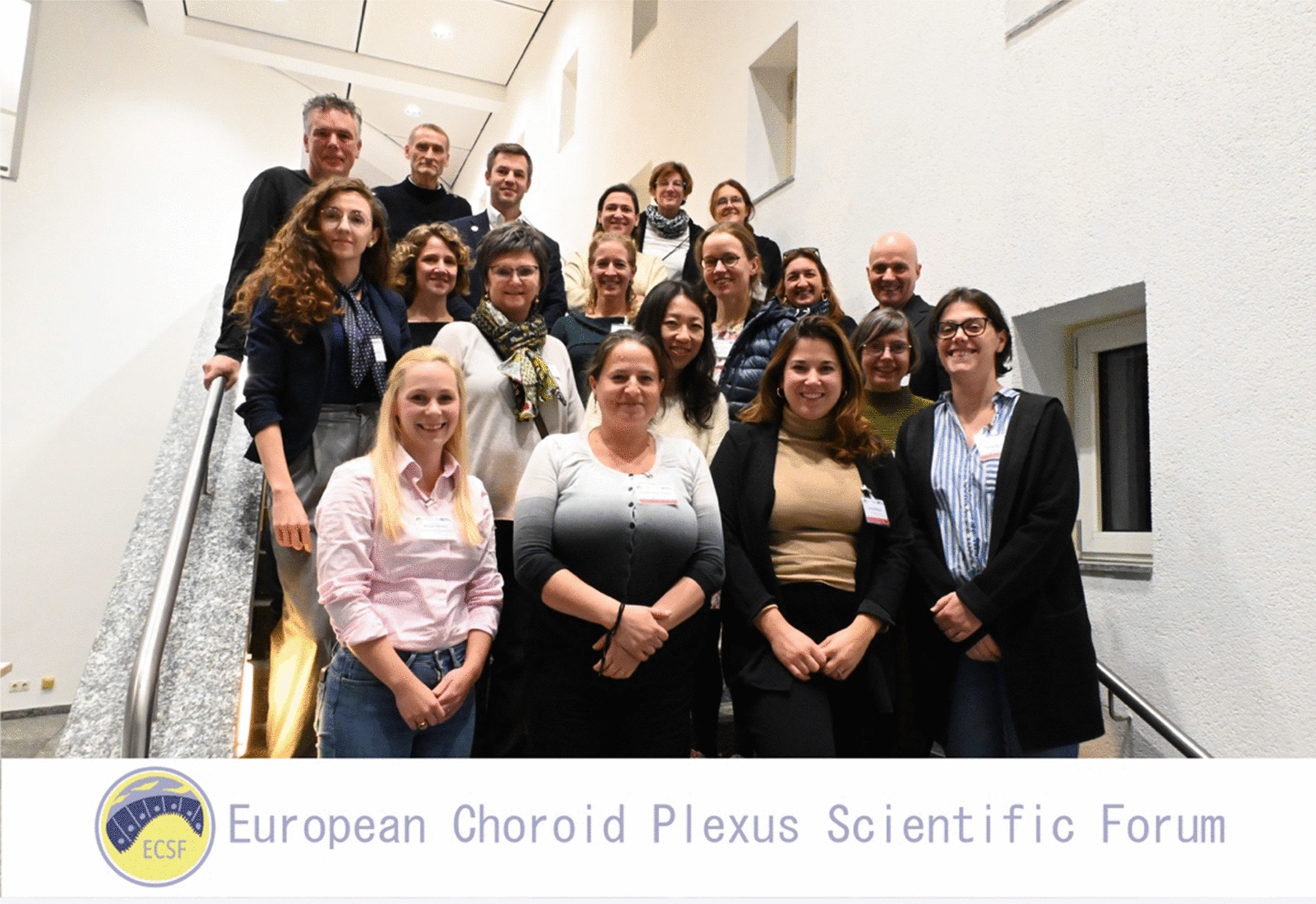
ECSF attendees, starting from the top, from left to right: Martin Hasselblatt, Jean-François Ghersi-Egea, Ross Paterson, Roosmarjin Vandenbroucke, Silvia Cappello, Nathalie Spassky, Aleksandra Deczkowska, Julia Wallmeier, Britta Engelhardt, Nanna MacAulay, Feng Quan, Nathalie Jurisch-Yaksi, Simona Lodato, Uwe Kordes, Denise Obrecht, Violeta Silva-Vargas*, Laura Pellegrini*, Fiona Doetsch and Annarita Patrizi*. Asterisk (*) indicates the organisers. Attendees not in the picture: Richard Gilbertson, Christian Hübner and Jeremy Wells. Heidelberg 7–9th November 2023

Many of the emerging concepts in the field have been recently discussed in a comprehensive review as well as illustrated in a ChP snapshot [11, 38]. The purpose of this article is to discuss some of the open questions in ChP and CSF biology, share a few examples of the groundbreaking discoveries presented at the ECSF meeting, and touch upon some of the future perspectives of this exciting and ever-growing field (Fig. 1).

## Choroid plexus cellular heterogeneity: a unique cytoarchitecture

**Fig. 2:**
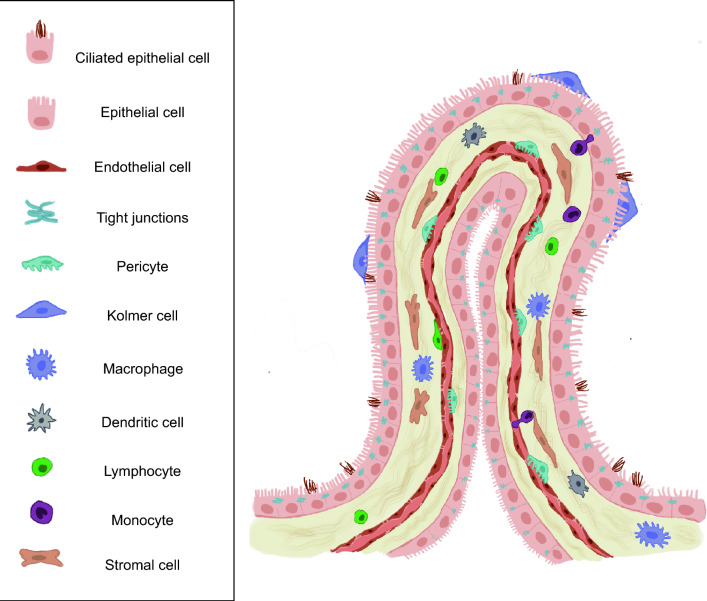
Schematic representation of the ChP cellular heterogeneity

The ChP extends in the third, lateral and fourth cerebral ventricles. It exhibits distinct regional identities [6], reflected in specific CSF proteomic profiles [22] and is a complex assembly of various cell types [6, 12, 38]. One of the key topics of discussion at the ECSF meeting was the intricate cellular and transcriptional diversity that distinguish each ChP (Fig. 2). The most prominent feature of the ChP is its layer of cuboidal epithelial cells. These cells are polarised and tightly connected by apical tight junctions, forming a continuous barrier, the blood-CSF-barrier. Their apical surfaces, facing the lumen of the ventricles, are highly convoluted and expanded with microvilli. This convoluted epithelial layer encapsulates the underlying stroma, rich with fibroblasts and immune cells such as macrophages, dendritic cells, and T cells. The intricate vascular network supporting the ChP is positioned within the stroma. The capillaries, composed by fenestrated endothelial cells, allow for the free flow of nutrients and immune cells extravasation from the blood to the ChP epithelial layer. The encapsulation of the stroma by the epithelial layer is not just structural but also functional. Historically, the epithelial cells have been mainly known for producing CSF and regulating its composition, their arrangement ensures an efficient interface with the blood supply in the stroma and circulating immune cells. Recent studies have started highlighting a more complex cellular composition of the ChP [5]. However, although we now appreciate more the complex cellular composition of the ChP, our understanding of the specific roles and functions within the ChP environment remains incomplete, highlighting the need for further in-depth research in this area.

Given the intricate cellular heterogeneity of the ChP, a key area of interest is the role and development of ciliated epithelial cells. Over time, the development and functionality of these unique ciliated cells undergo changes, which are crucial for maintaining cerebral homeostasis. Understanding cilia development and alterations over time is critical for comprehending how the ChP adapts and functions throughout different life stages. Recent studies shedding light on the nodal-like cilia of the ChP and their changes over time, revealed how these cells adapt throughout different life stages in mice [13]. The ChP epithelial cilia are distinct from the longer and beating ependymal cell cilia, lining the ventricle and spinal cord’s central canal, which contribute to the directional flow of CSF across the ventricles [42]. Interestingly, these features may be mammalian specific, as ChP epithelial cells in zebrafish harbour beating cilia capable of generating fluid flow [7, 16, 17]. During development and aging, ChP epithelial cells undergo complex morphological transformations, including the regression of the ciliary axonemes over time, shortening of microvilli and overall cell flattening in mice. Elongation of mitochondria shape was also observed with aging [39]. Zebrafish ChP cells also undergo complex morphological transformation during development with the acquisition of a multiciliated fate [7, 16]. Altogether, these changes occurring during lifespan could have profound implications for understanding age-related alterations in CSF circulation and their link to various neurological conditions. Clinically, this research is particularly relevant in the context of ciliopathies, such as de novo mutations in *FOXJ1* resulting in motile ciliopathy characterised by hydrocephalus and airways defects [47, 48]. Disruption of normal ChP multiciliation also underlies some forms of ChP tumorigenesis [20]. Ciliopathies have far-reaching implications, with potential impacts on brain health and function.

The ChP stroma has received little attention in the past but is now becoming a central point of focus in the ChP field. The stroma consists of a rich mix of cells, including endothelial cells, pericytes, fibroblasts, macrophages, dendritic cells, lymphocytes among others. Intriguingly, macrophage can be located either in the stroma (stromal macrophages) or along the apical surface of the ChP epithelial cells (Epiplexus Kolmer cells), closely associated with the microvilli border. The diversity and functions of all of these cellular components is currently being investigated, and new insights suggest that most of these cell types express unique markers. Some of these cells, including the endothelial cells, have the potential of acting as barrier structures in response of inflammatory stimuli [2]. This cellular diversity underlines the stroma’s multiple roles, ranging from supporting the epithelium, to playing a central role in immune response within the central nervous system (CNS) [4, 19, 36]. Several ongoing studies have shown that ChP immune cells are actively involved in neuroinflammatory conditions, influencing the entry and regulation of immune cells into the CNS and the CSF.

### Open questions

1. How can we better understand how the unique cell types found in the ChP, such as the epithelial and stromal cells, emerge and undergo distinct cell states, and what is their individual function and their interaction within the tissue? How do ChP epithelial cells communicate with the stroma and vice versa? Determining how these cells individually and collectively contribute to the ChP role in CSF secretion, barrier maintenance and CNS immune surveillance is a key open question.
2. How are the different immune cells recruited to and traverse the ChP stroma and epithelial compartments, especially under pathological conditions? For example, the cellular machinery at the apical or CSF side of the epithelial cells has received a lot of attention in the past but the cellular mechanisms happening at the basal side of the epithelium and at the endothelium are far less understood.
3. How conserved is this cellular heterogeneity and its impact on the ChP and CSF development and function across species?

### New methods and future perspectives

One of the future perspectives discussed at the ECSF meeting focused on the ambitious project of building a comprehensive ChP cell atlas. This initiative aims to map the diverse array of cell types within each ChP, a task complicated by the heterogeneity of these cells. The discussion also explored how cells are distributed within the ChP in comparison with other epithelial tissues. It was noted that, unlike in organs like the gut where cell differentiation follows a more predictable path, the ChP presents a different picture, with a less linear differentiation pattern. Promising machine learning approaches combining multi-omics datasets will become more and more necessary to unravel the local cellular complexities [1]. For example, integrating single-cell RNA sequencing with proteomic analysis and spatial mapping will be instrumental in deciphering the intricate cellular characteristics, distribution patterns, and identities within the ChP [21].

To improve our understanding of the cellular heterogeneity within the ChP, advanced whole tissue or ultrastructural imaging techniques coupled with automated analysis and segmentation are being developed. One example is combining wide field and confocal microscopy with EM, coupled with RNA labelling, or the integration of single-cell and spatial transcriptomics in cross sections with spatial maps of the entire ChP whole mounts. Combining these various strategies will result in a more precise and comprehensive visualization of subcellular structures and specific expression patterns allowing to build spatial distribution maps of distinct cell subtypes, leading to a deeper and more nuanced understanding of the complex cellular environment within the ChP.

Because of its multiple locations within the brain and its unique cellular architecture, the ChP represents a key integration hub, responding to multiple stimuli with the goal of constantly maintaining brain homeostasis. These responses to peripheral stimuli, which will be discussed in the following section, include immune cell trafficking, the exchange of nutrients necessary for CSF production, the upregulation or downregulation of specific types of secretion, the removal of waste products from the CSF and the regulation of barrier dynamics. Understanding the heterogeneity of all cellular components and how they uniquely contribute to the ChP function would be critical to connect its anatomical structure with its physiological roles in the brain.

### The choroid plexus, a central and dynamic regulator of brain environment

In recent years, advancements in our understanding of CSF production have challenged the traditional osmotic models, highlighting a more complex and regulated process [44]. In particular, the simple model of CSF production via osmotically driven transport of water through aquaporin 1 (AQP1) channels has been challenged by the observation that AQP1 knock-out mice only show a 20% reduction in CSF production [28] and that the ChP has the ability to secrete CSF even against an oppositely directed osmotic gradient [27]. Central to this paradigm shift is the role of the water-translocating cotransporter Na + /K + /2Cl- (NKCC1) in the ChP epithelium. NKCC1's critical role in CSF production is evidenced by a significant reduction in CSF secretion when the channel is blocked [44].

CSF plays a multifaceted role in brain function and development and ChP’s plasticity is key in shaping the dynamic composition of CSF [10]. For example, this plasticity is reflected in CSF adaptation during various physiological states and its influence on the regulation of neural stem cells [41]. CSF secretion and composition exhibits variations during day and night [8, 43].The ChP is instrumental in this regulation, functioning as a circadian gatekeeper that modulates CSF composition in response to circadian rhythms [9, 26, 34]. It is therefore important to better understand how the ChP regulate CSF composition and how these processes could go wrong in certain diseases. ChP circadian dysfunction could disrupt the related sleep–wake cycles and ultimately affect metabolic waste clearance.

CSF composition can vary depending on sex and age [14, 35]. Young CSF has been shown to restore memory function in aged mice by promoting oligodendrocyte progenitor proliferation [14]. Sex dimorphism in the ChP and CSF have also been reported. The ChP is known to express sex hormone receptors [37], and differences between males and females are currently being investigated at the transcriptomic and proteomic level. These differences are also reflected in changes in ChP function between the sexes, including variations in the secretion of CSF proteins and hormones, barrier function, immune responses, and the elimination of toxic substances. These differences are significant for maintaining normal brain function and may underlie important disease mechanisms across sexes. The intricate balance of these functions is vital for maintaining brain health.

Finally, the CSF also contains a significant concentration of extracellular vesicles (EVs), whose composition and physiological functions are still being unraveled. Recent advancements, are exploring how EVs within the CSF may facilitate cellular communication within the brain, promoting neuronal growth and synaptic function [32]. Understanding these processes is an emerging field that could reveal new insights into brain health and diseases and could be exploited as a potential strategy for drug delivery.

## Immune signals and microbial products shape brain development via choroid plexus

The ChP is a central hub linking the peripheral environment with the CNS and influencing brain development particularly through interactions with immune signals and microbial products. Examples of ChP triggers are bacterial lipopeptides, amyloid beta, and short-chain fatty acids (SCFA); these molecules can significantly impact the production of EVs by the ChP and affect both epithelial and endothelial barrier function [3, 23, 40]. Experiments conducted in germ-free mice devoid of microbiome show ChP barrier defects which can be rescued with addition of SCFA [49]. These findings may offer new insights into the gut-brain axis via ChP regulation. The role of cytokines is also gaining attention, especially during perinatal stages, when the brain gets into contact with pathogens and microbes. Pioneering work revealed that the ChP has a critical role in neuroprotection and detoxification linked to the expression, especially high during postnatal development, of antioxidant and xenobiotic-conjugating enzymes conjugating xenobiotics such as sulfotransferases (SULT) and glutathione-S-transferases (GST) [18, 45]. These enzyme families work in concert with the ChP efflux transporters to limit entry of toxic compounds and may participate to the control of postnatal neuroinflammation driven by microbial elements in the brain. In the future, it will be essential to understand how microbe colonisation affects the immune system of the ChP and thereby shaping the brain development. In summary, there is a complex and dynamic relationship between the ChP, immune signals, metabolizing enzymes and microbial elements that is critical for shaping brain health and development.

### Open questions

1. The ChP has multiple functions: barrier properties, CSF production, polypeptide secretion, immune surveillance, detoxification, however we usually study them in isolation. Is it possible to have a holistic view of the multiple ChP functions?
2. What is the impact of the microbiome on the development and maturation of the ChP and how does the ChP respond to changes in the environment over time?

### New methods and future perspectives

Due to a lack of ChP specific genetic driver lines, it has been challenging to specifically target the ChP until now [46]. The repertoire of tools to study the ChP is getting larger, and several techniques were discussed at the ECSF. The ablation of ChP in zebrafish using nitroreductase (NTZ) can be used to look at the impact on the brain ventricular system [16]. Genetic tools such as AAV2/5, administered by intracerebroventricular injection, selectively transduce the ChP epithelial cells allowing for chronic and acute manipulation [25]. Finally, imaging techniques such as TEM, light-sheet and two-photon microscopy offer invaluable insights into the intricate dynamics among ChP epithelial cells, stromal cells, and the immune cells lining the barrier. These advanced methods are instrumental in determining the role of the ChP in immune cell invasion into the brain and the complex interactions with other resident immune cells. Understanding these dynamics is crucial for unraveling the mechanisms of immune surveillance and response within the CNS.

## From laboratory to clinic and back: integrating fundamental research and clinical insights

The need for integrating fundamental research with clinical findings is becoming clearer, and emerging models, such as ChP organoids, represent an exciting opportunity to bridge this gap as they allow us to explore human biology in a complex 3D cellular microenvironment. A significant challenge persists to generate models that accurately mimic the function and interaction between multiple cell types, including for example, vascular and immune cells. The use of state-of-the-art protocols, that combine multiple cell types within one organoid, is critical to shed light on the communication between ChP cells, both in health and disease. ChP organoids have now proven useful for several applications including modeling human-specific drug permeability, pathogen entry and CSF secretion [30, 31]. The ChP is a target for several pathogens including SARS-CoV-2. SARS-CoV-2 infection and barrier damage was shown in ChP organoids [15, 31] and these findings were demonstrated also in adult Covid patients [50] as well as in fetal brain tissue associated with infection [24]. ChP organoids incorporating microglia have also been used to model HSV-1 infection [33]. New exciting use of this technology has been presented at ECSF, as organoids infected with other pathogens such as *S.suis* or organoids employed to measure the turnover of CSF proteins in neurodegenerative diseases. A targeted mass spectrometry approach was used to quantify protein turnover in CSF-like fluid from organoids, this method could be used to interrogate how defects in CSF turnover resulting in CSF stagnation can lead to neurodegeneration. Organoids can also be a useful tool to model monogenetic diseases, for example mutations in ciliary genes linked to hydrocephalus [47]. Because organoids can be created via the genetic reprogramming of somatic cells from patients, thereby preserving the unique genetic background of an individual, they represent a powerful tool for studying complex genetic diseases.

One of the ECSF focuses was to prioritise research related to choroid plexus tumors (CPT), rare but potentially devasting diseases, with a peak incidence of choroid plexus carcinoma in early infancy posing a particular clinical challenge. These tumors are a distinct and complex type of brain neoplasm, highlighting the need for an in-depth exploration of their origins, treatment approaches, and effects on the CNS. Delineating the cellular origins of ChP tumors, whether from epithelial cells or adjacent lineages, is crucial. Organoids can be used to investigate the molecular diversity of tumors of the ChP and to refine current therapeutic strategies to specifically target tumor cells. Additionally, assessing the impact of these tumors on CSF dynamics and neurological function is vital and there is very little known at the moment about these dynamics. At the meeting, we delved into specific challenges associated with studying ChP tumors. Among the key issues highlighted were the difficulties clinicians encounter due to the rarity of these conditions. This rarity is a challenge for clinical trialists and international treatment and guidance consensus is mainly to be addressed by global co-operation. Outcome may be compounded by haemorrhagic complication, hygroma and CSF circulation or the aggressive nature of choroid plexus carcinoma, which unfortunately often results in poor survival rates.

### Open questions

1. What are the functional consequences of malfunction of the ChP? Are there potential therapeutic targets within the ChP that could be exploited to modulate brain function?
2. How might an improved understanding of ChP function open new avenues for drug delivery to the central nervous system?
3. What are the most effective strategies for engaging with patients to enhance their clinical outcomes and simultaneously facilitate the collection of samples for research purposes?

### New methods and future perspectives

The recent advancements in research methodologies have significantly enhanced our understanding of ChP diseases. Notably, the application of the Stable Isotope Labelling Kinetic (SILK) method [29] in patients with normal pressure hydrocephalus has been pivotal in monitoring changes in the dynamics of CSF biomarkers, such as Transthyretin (TTR). Furthermore, the development of ChP organoids that can reach mature stages offers an innovative way to mimic CSF secretion and replicate the protein turnover dynamics of disease-relevant biomarkers. Lastly, the exploration of new techniques to target this barrier for drug delivery, particularly through investigating mechanisms of transcytosis, is underway. This research holds great promise for yielding less invasive and more effective treatments, leveraging the ChP's strategic location and its influential role in CSF signaling. These developments mark a significant step forward in the pursuit of advanced treatments for ChP-related conditions.

Another key point discussed is how to increase patient involvement in choroid plexus tumor research. Due to the rarity of this tumor entity, a few strategies were discussed, such as the establishment of an interdisciplinary research networks and an international platform to coordinate and organise patients worldwide expanding the, already existing, International SIOP-CPT Registry coordinated by Dr. med. Denise Obrecht, at the University Medical Centre Hamburg-Eppendorf. The SIOP-CPT registry is involved with the choroid plexus carcinoma guidance (lead by Dr. Jenny Adamski; SIOPE-CPT-BTG) within the European Reference Network for Paediatric Cancer diseases (ERN PaedCan) and the PNOC-CPT trial plans (lead by Prof. Dr. Sabine Müller) It is important to devise a multimodal approach to ensuring effective coordination with patients for improved clinical outcomes and sample collection. Patient education, involvement and constant communication are crucial throughout the process, from the beginning by giving rigorous ethical consent, to patient support, and follow-up, including updates on research progress. Together these can foster a sense of contribution and improve clinical engagement always complying with legal and ethical standards. Finally, establishing a feedback mechanism for patients to express concerns or inquire about the research would also enhance trust and transparency. These strategies, discussed during the meeting, collectively could create a robust framework for engaging patients in ChP tumor research, thereby enhancing both clinical outcomes and the quality of scientific inquiry.

## Conclusion

In this short commentary we were not able to discuss many other emerging topics around ChP and CSF research. It is certainly an exciting moment in this rapidly expanding field, however, despite the increasing accumulation of knowledge on the ChP-CSF biology, several gaps remain, emphasising the importance of facilitated communication and interaction between researchers from divergent institutions and disciplines. A major strength of the ECSF was the open discussions, sharing of ideas, commitment to a more diverse and equitable scientific environment and encouragement of cooperative endeavors among the scientific community. In conclusion, we hope this to be the first of a long series of fruitful meetings that will strengthen the community and ultimately lead to uncover the secrets of this fascinating tissue of the brain.

## Data Availability

Not applicable.

## Abbreviations

ECSF: European Choroid plexus Scientific Forum
ChP: Choroid plexus
CSF: Cerebrospinal fluid
FOXJ1: Forkhead box protein J1
CLEM: Correlative light and electron microscopy
EM: Electron Microscopy,
AQP1: Aquaporin-1,
NKCC1: Na^+^/K^+^/2Cl^−^ cotransporter
EV: Extracellular vesicle
SCFA: Short-chain fatty acids
SULT: Sulfotransferases
GST: Glutathione-S-transferases
NTZ: Nitroreductase
TEM: Transmission electron microscopy
3D: Three dimensional
SARS-CoV-2: Severe acute respiratory syndrome coronavirus 2
HSV-1: Herpex simplex virus-1
CPT: Choroid plexus tumor
SILK: Stable isotope labelling kinetic
TTR: Transthyretin
